# Oxidative Alteration of Trp-214 and Lys-199 in Human Serum Albumin Increases Binding Affinity with Phenylbutazone: A Combined Experimental and Computational Investigation

**DOI:** 10.3390/ijms19102868

**Published:** 2018-09-21

**Authors:** Luiza de Carvalho Bertozo, Ernesto Tavares Neto, Leandro Cristante de Oliveira, Valdecir Farias Ximenes

**Affiliations:** 1 Department of Chemistry, Faculty of Sciences, UNESP–São Paulo State University, Bauru, SP 17033-360, Brazil; luiza_bertozo@yahoo.com.br; 2 Department of Physics–Institute of Biosciences, Humanities and Exact Sciences, UNESP–São Paulo State University, São José do Rio Preto, SP 15054-000, Brazil; e.tavares@unesp.br (E.T.N.); leandro.cristante@unesp.br (L.C.O.)

**Keywords:** albumin, oxidative stress, taurine dibromamine, tryptophan, *N*′-formylkynurenine, phenylbutazone

## Abstract

Human serum albumin (HSA) is a target for reactive oxygen species (ROS), and alterations of its physiological functions caused by oxidation is a current issue. In this work, the amino-acid residues Trp-214 and Lys-199, which are located at site I of HSA, were experimentally and computationally oxidized, and the effect on the binding constant with phenylbutazone was measured. HSA was submitted to two mild oxidizing reagents, taurine monochloramine (Tau-NHCl) and taurine dibromamine (Tau-NBr_2_). The oxidation of Trp-214 provoked spectroscopic alterations in the protein which were consistent with the formation of *N*′-formylkynurenine. It was found that the oxidation of HSA by Tau-NBr_2_, but not by Tau-NHCl, provoked a significant increase in the association constant with phenylbutazone. The alterations of Trp-214 and Lys-199 were modeled and simulated by changing these residues using the putative oxidation products. Based on the Amber score function, the interaction energy was measured, and it showed that, while native HSA presented an interaction energy of −21.3 kJ/mol, HSA with Trp-214 altered to *N*′-formylkynurenine resulted in an energy of −28.4 kJ/mol, and HSA with Lys-199 altered to its carbonylated form resulted in an energy of −33.9 kJ/mol. In summary, these experimental and theoretical findings show that oxidative alterations of amino-acid residues at site I of HSA affect its binding efficacy.

## 1. Introduction

Oxidative stress occurs from the imbalance between the endogenous production and neutralization of reactive oxygen species (ROS) [1,2]. The excess of ROS causes oxidative damage in tissues and biomolecules. These molecular alterations are associated with the development of chronic and degenerative diseases, such as cancer, neurological diseases, and cardiovascular diseases, among others [3,4,5,6]. As well established, proteins are among the most susceptible biomolecules in an oxidizing environment. This is due to the presence of oxidizable amino-acid residues, such as tryptophan, tyrosine, methionine, and cysteine, among others. Their oxidations are linked to alterations in the secondary and tertiary structure of proteins, modifying their form and their function [7]. In fact, there is evidence that protein oxidation is associated with initiation and progression of various diseases [8]. For instance, modifications of brain proteins due to oxidation were correlated with alterations in biochemical processes in Alzheimer’s [7] and Parkinson’s diseases [9]. In diabetes mellitus, the oxidative environment caused by abnormal metabolism was linked to the physiopathology of this devastating disease. Particularly important in diabetes is the decrease in the antioxidant capacity of human serum albumin (HSA) the main constituent protein of blood plasma [10,11].

HSA is a multifunctional carrier protein. It is capable of transporting exo- and endogenous compounds such as hormones, fatty acids, and drugs. HSA is composed of 585 amino-acid residues, where 35 are cysteine residues, forming 17 disulfide bonds, which contribute to protein stability and flexibility, and the Cys-34 residue is the only free sulfhydryl group [12]. In addition to cysteine, HSA has only one tryptophan residue, which, in addition to being highly susceptible to oxidation, is the main residue responsible for this protein’s intrinsic fluorescence when excited at 295 nm [13,14]. The structure of the HSA is organized into homologous domains (I, II, and III), and each domain has two subdomains (A and B) [15]. HSA has two major binding sites known as Sudlow site I and Sudlow site II, which are located in subdomains IIA and IIIA, respectively [16]. It is worthy of note that the only tryptophan residue in HSA is located at site I and contributes to drug stabilization in this site [17].

As the most abundant protein in blood plasma, HSA is constantly exposed to an oxidative stress environment [18]. In blood plasma, the oxidized and reduced forms of HSA are present. The reduced form is due to free Cys-34. In normal physiological conditions, the reduced form of the protein (70–80%) is predominant [19,20]. The decrease in concentration of reduced HSA is reported in several diseases where oxidative stress is an “author” of their physiopathology, such as renal complications, diabetes, and liver diseases [21,22,23]. The structural alteration and blood concentration of HSA are also important issues during the administration of high-affinity drugs, since they can impact drug efficiency [21]. It was shown that the modification of amino-acid residues in HSA by oxidation may influence the ability of the protein as a drug carrier, particularly in multi-therapies. For instance, the alteration of the association constant (Ka) between drugs and oxidized albumin was observed in the co-administration of sulfasalazine and sulindac [24]. A similar result was observed in the evaluation of the interaction capacity between oxidized albumin and methotrexate used in the treatment of rheumatoid arthritis [25]. Patients with hepatic insufficiency presented increased oxidized albumin fractions and a consequent change in the binding capacity of the protein; in this case, dansylsarcosine, a classic site II ligand, was used [26].

Due to the importance of albumin as a drug carrier and the evidence that its oxidation is able to alter this essential function, in this work, we aimed to promote an efficient alteration of the amino-acid residues most susceptible to oxidation, without, however, causing a significant degradation of HSA. To accomplish this task, two mild oxidants, taurine chloramine (Tau-NHCl) and taurine dibromamine (Tau-NBr_2_) were employed. Phenylbutazone, a site I drug [27], was used to evaluate the alteration in the association constants before and after the oxidation. The experimental results were correlated with the stabilization energy obtained by molecular docking simulation of the native and computationally modified protein.

## 2. Results

### 2.1. Efficacy of Tau-NBr_2_ as an Oxidant of Free Aromatic Amino Acids

Tau-NBr_2_ was used as a relatively mild and efficient oxidant of tryptophan in proteins [28]. Here, this chemical feature of Tau-NBr_2_ was initially demonstrated by studying the oxidation of *N*-acetyl tryptophan (*N*acTrp) and the inhibitory effects provoked by the addition of other amino acids that constitute the binding site I of HSA. To highlight the relative high reactivity of Tau-NBr_2_ with tryptophan, a comparison was also made with the tyrosine derivative, *N*-acetyl tyrosine (*N*acTyr). Although methionine and the reduced form of cysteine are not usually described as relevant for the complexation of ligands in HSA [17,29,30,31], they were also investigated as inhibitors, since these amino acids are highly susceptible to oxidation in proteins [10].

**Figure 1:**
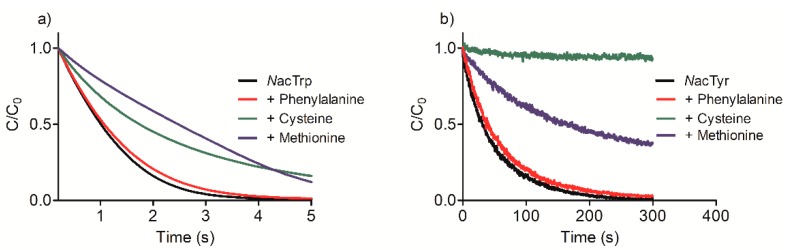
Fluorescence decay due to oxidation of (**a**) *N*-acetyl tryptophan (*N*acTrp) and (**b**) *N*-acetyl tyrosine (*N*acTry) by taurine dibromamine (Tau-NBr_2_) and the inhibitory effect of amino acids. Reaction conditions: *N*acTrp or *N*acTyr, 50 µmol·L^−1^; Tau-NBr_2_, 500 µmol·L^−1^ in phosphate-buffered saline (PBS); pH 7.4 at 25 °C. When present, the amino acids were 500 µmol·L^−1^.

Figure 1a shows that *N*acTrp was promptly oxidized by Tau-NBr_2_, confirming the efficacy of this chemical in depleting tryptophan and its derivatives. Figure 1a also shows the effect of the presence of phenylalanine in the kinetic profile of *N*acTrp consumption. As can be seen, even though a 10-fold molar excess was used, the inhibition of *N*acTrp oxidation was insignificant. As expected due to their low oxidability, the addition of others amino acids involved in the complexation of ligands at site I, such as leucine, alanine, arginine, lysine, and serine, was also ineffective in the inhibition of oxidation of *N*acTrp (results not shown). The exceptions were methionine and cysteine, which were able to reduce the efficiency of oxidation. However, at 10-fold molar excess in relation to *N*acTrp, these amino acids were only able to decrease the rate of oxidation, but not the consumption of *N*acTrp.

**Figure 2:**
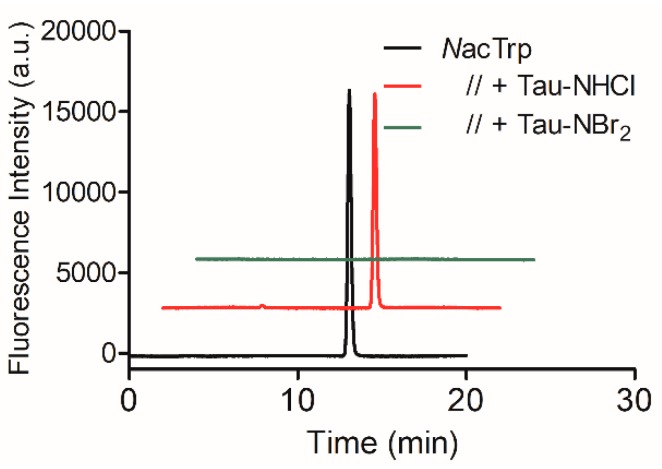
Chromatography profile of *N*acTrp consumption by Tau-NBr_2_ and taurine chloramine (Tau-NHCl). Reaction conditions: *N*acTrp, 50 µmol·L^−1^; Tau-NBr_2_, 500 µmol·L^−1^ in PBS; Tau-NHCl, 1000 µmol·L^−1^ in PBS; pH 7.4; incubation for 1 h at 25 °C.

The tyrosine derivative *N*acTyr was much less reactive compared to *N*acTrp. Figure 1a,b show that, while *N*acTrp was depleted in less than five seconds, *N*acTyr took about 300 s. Another difference was the inhibitory effect of the competitor amino acids. In this case, the consumption of *N*acTyr was partially inhibited by 10-fold molar excess of methionine, and totally inhibited by cysteine (Figure 1b). *N*acTrp was also submitted to oxidation by Tau-NHCl, which is much less reactive than Tau-NBr_2_ [32]. For this reason, the reactions were conducted for 1 h and the remaining amino acids were measured by HPLC. The results depicted in Figure 2 confirmed the expectation, since Tau-NHCl was a much less efficient oxidant of *N*acTrp compared to Tau-NBr_2_.

### 2.2. Oxidation of Amino-Acid Residues in HSA by Tau-NBr_2_ and Tau-NHCl

**Figure 3:**
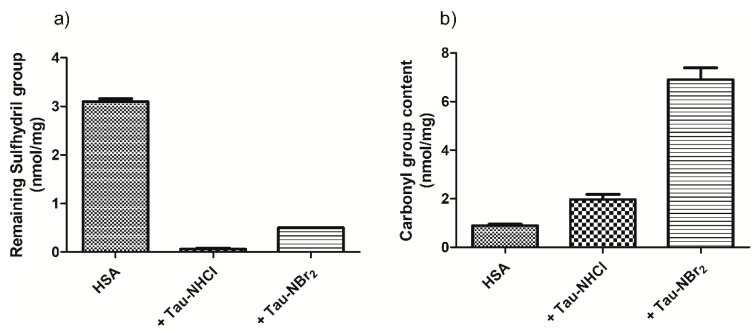
Tau-NBr_2_ versus Tau-NHCl: (**a**) oxidation of sulfhydryl residues, and (**b**) formation of carbonyl groups in human serum albumin (HSA). Reaction conditions: HSA, 50 µmol·L^−1^; Tau-NBr_2_, 500 µmol·L^−1^ in PBS; Tau-NHCl, 1000 µmol·L^−1^ in PBS; pH 7.4; incubation for 1 h at 25 °C. See experimental section for details of the methods for measurement of the remaining sulfhydryl and the formation of carbonyl groups.

A confirmation that both Tau-NBr_2_ and Tau-NHCl provoked the depletion of sulfhydryl residues in HSA is shown in Figure 3a. In these experiments, the oxidants were used at 10- to 20-fold molar excess to the protein, which was enough to completely deplete the aromatic amino acids, as demonstrated later. As shown, both oxidants were able to deplete the sulfhydryl residues in HSA, with Tau-NHCl being more effective. On the other hand, the opposite result was obtained regarding the generation of protein carbonyl groups. Figure 3b shows the amount of carbonyl groups produced in HSA oxidized by Tau-NHCl and Tau-NBr_2_, with the latter being more efficient.

**Figure 4:**
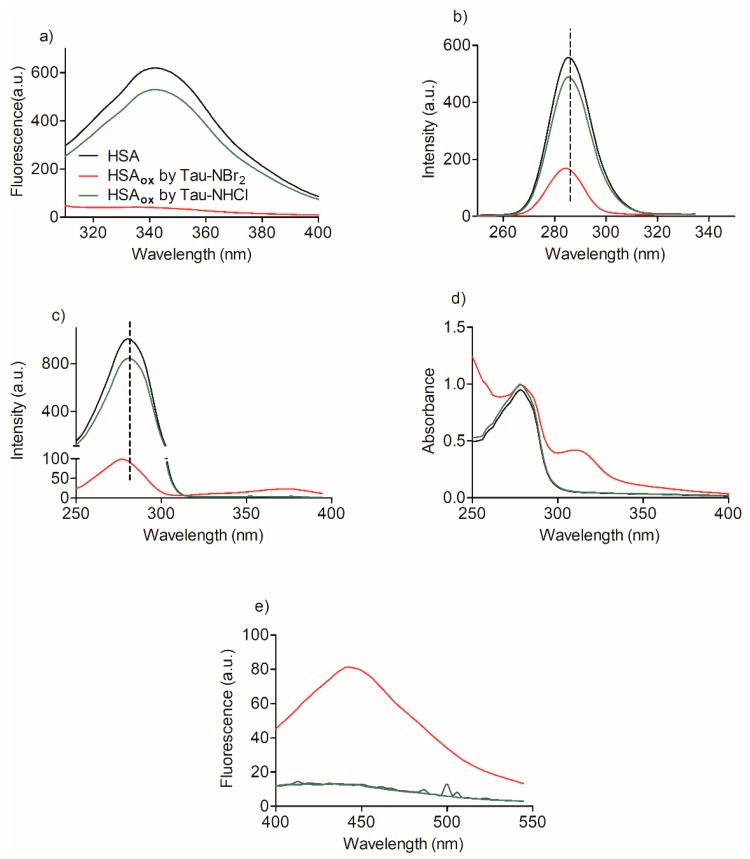
Spectral alteration in HSA provoked by oxidation. (**a**) Intrinsic fluorescence spectra by excitation at 295 nm. (**b**) Synchronous fluorescence at ∆λ 15 nm. (**c**) Synchronous fluorescence at ∆λ 60 nm. (**d**) Absorbance spectra. (**e**) Fluorescence spectra by excitation at 320 nm. Reaction conditions: HSA, 10 µmol·L^−1^; Tau-NBr_2_, 100 µmol·L^−1^ in PBS; Tau-NHCl, 200 µmol·L^−1^ in PBS; pH 7.4; incubation for 1 h at 25 °C.

The capacity of Tau-NBr_2_ as an efficient oxidant of the tryptophan residue in HSA can be observed by analyzing the results in Figure 4. Firstly, the intrinsic fluorescence of HSA, which is mainly related to tryptophan when excited at 295 nm [33], was completely depleted by Tau-NBr_2_, while Tau-NHCl was significantly less effective. Similar results were obtained when studying the synchronous fluorescence of HSA. This spectroscopic technique can detect alterations in the microenvironments of tyrosine and tryptophan residues in proteins [34]. Figure 4b shows that, in addition to the depletion of the band, a small blue shift (~2 nm) was observed using Tau-NBr_2_. The alteration was still more pronounced upon fixing the delta wavelength at 60 nm. This result confirms the higher efficacy of Tau-NBr_2_ as an oxidant of tryptophan when compared to tyrosine. In this case, in addition to the depletion and blue shift (~4 nm), a new band was detected at 375 nm (Figure 4c). Confirming the previous results, Tau-NHCl was much less efficient.

The appearance of a new band at 375 nm in the synchronous fluorescence experiment indicated that a new chromophore was formed using Tau-NBr_2_ as an oxidant. This finding was confirmed by measuring the alteration in the absorbance spectrum (Figure 4d) and also the appearance of a new fluorescent band by excitation at 315 nm (Figure 4e). These spectroscopic alterations in HSA were additional evidence that tryptophan is a target of Tau-NBr_2_, because they are consistent with the formation of *N*′-formylkynurenine, the product of oxidative cleavage of the indole ring of tryptophan. This degradation product is usually detected in proteins by its spectroscopic characteristics, i.e., absorption around 320 nm and fluorescence at 450 nm [35,36].

**Figure 5:**
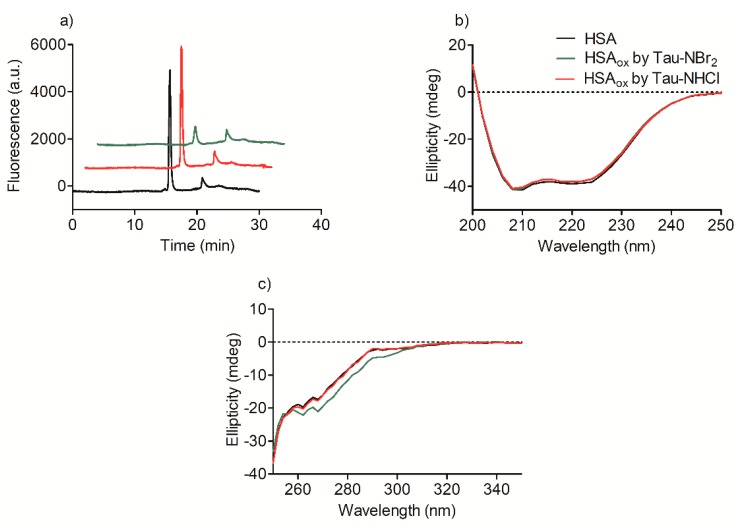
HPLC profile and alterations in the circular dichroism (CD) spectra of HSA provoked by oxidation. (**a**) Alteration in HPLC peak profile. Reaction conditions: HSA, 10 µmol·L^−1^; Tau-NBr_2_, 100 µmol·L^−1^ in PBS; Tau-NHCl, 200 µmol·L^−1^ in PBS; pH 7.4; incubation for 1 h at 25 °C; fluorescence detector set at 295/340 nm. (**b**) Far-ultraviolet (UV) CD, and (**c**) near-UV CD spectral alteration. Reaction conditions: HSA, 30 µmol·L^−1^; Tau-NBr_2_, 300 µmol·L^−1^ in PBS; Tau-NHCl, 600 µmol·L^−1^ in PBS; pH 7.4; incubation for 1 h at 25 °C. For the far-UV CD analysis, the reaction mixture was diluted to 2.0 µmol·L^−1^ before measurements.

To evaluate whether a more profound alteration could be taking place and leading to aggregation or fragmentation of the protein, its reverse-phase chromatographic profile was also studied. The use of a fluorescence detector in the HPLC system indicated a decrease in the peak intensity for the protein oxidized by Tau-NBr_2_, which is consistent with the previous results (Figure 5a). However, no significant difference was observed in the retention time or peak shape, which is an indication that the protein was not fragmented or aggregated [37]. Similar experimental evidence was obtained by analyzing the far-ultraviolet (UV) circular dichroism (CD) spectra of the protein before and after oxidation. As can be seen, no alteration was detected in this region of the spectrum, which suggests that the secondary structure of the protein was not significantly altered (Figure 5b). However, in the near-UV CD region, which is linked to the absorption of aromatic and sulfhydryl residues [38], Tau-NBr_2_ again showed its efficiency as an oxidant (Figure 5c), a result that is in agreement with those obtained by fluorescence.

### 2.3. Alteration in the Binding of Phenylbutazone Provoked by Oxidation of HSA

**Figure 6:**
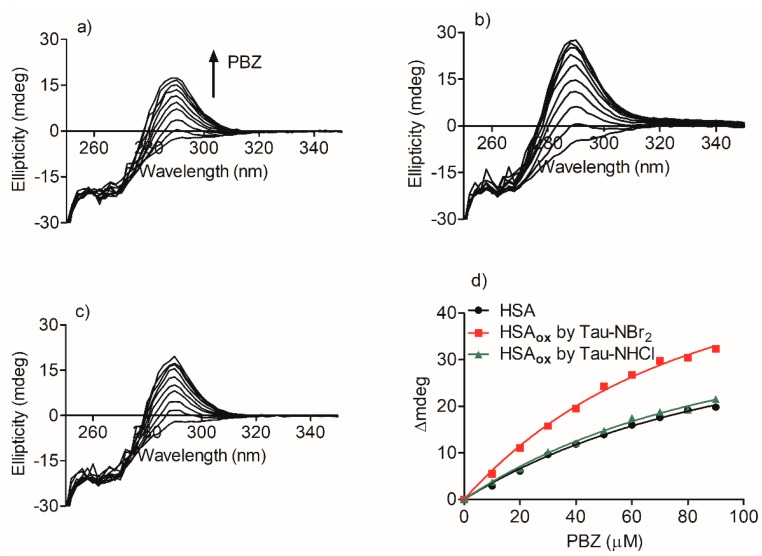
Determination of association constant (Ka) between HSA and phenylbutazone (PBZ). Induction of chirality in PBZ upon its binding to (**a**) native HSA, (**b**) HSA oxidized by Tau-NBr_2_, and (**c**) HSA oxidized by Tau-NHCl. (**d**) Curve fitting of experimental data (∆CD at 292 nm) using Equation (1) for determination of association constants. Values of Ka obtained were 1.1 × 10^4^ M^−1^ (*r*^2^ = 0.9996) for HSA, 1.7 × 10^4^ M^−1^ (*r*^2^ = 0.9649) for HSA oxidized by Tau-NBr_2_, and 1.2 × 10^4^ M^−1^ (*r*^2^ = 0.9991) for HSA oxidized by Tau-NHCl.

The association constant between oxidized HSA and phenylbutazone was measured and compared with the values obtained for the native protein. The method for determining the constants was based on the generation of induced circular dichroism (ICD) in phenylbutazone, provoked by its binding to HSA. Figure 6a shows the generation of a positive ICD band centered at 292 nm and its increase until saturation by adding phenylbutazone at a fixed concentration of HSA. The ICD increase provoked by the addition of phenylbutazone (Figure 6d), and the application of the rationalization and mathematical derivatization presented by Zsila (Equation (1)) resulted in the association constant (Ka) between HSA and phenylbutazone being determined. In this equation, the intensity of the ICD signal (θ, mdeg) was measured against the concentration of the ligand (phenylbutazone, L), by keeping the concentration of protein (P) constant [39].

θ (mdeg) = K/2(P + L + (1/K_a_) − sqrt(P + L + (1/K_a_))^2^ − 4PL) (1)

The same approach was used to calculate the association constant for oxidized HSA. In these experiments, before the titration with phenylbutazone, the samples were incubated for 10 min with methionine, which was used to scavenge the excess Tau-NBr_2_ and Tau-NHCl used in the oxidation. Figure 6b,d show that the previous oxidation by Tau-NBr_2_ caused a significant increase in the association constant between HSA and phenylbutazone. On the other hand, the oxidation of HSA by Tau-NHCl did not result in an alteration compared to the native protein (Figure 6c,d). The association constants obtained were 1.1 × 10^4^ M^−1^ (*r*^2^ = 0.9996) for HSA, 1.7 × 10^4^ M^−1^ (*r*^2^ = 0.9649) for HSA oxidized by Tau-NBr_2_, and 1.2 × 10^4^ M^−1^ (*r*^2^ = 0.9991) for HSA oxidized by Tau-NHCl.

### 2.4. Computational Alteration of Trp-214 and Lys-199 and Molecular Docking Simulation

**Figure 7:**
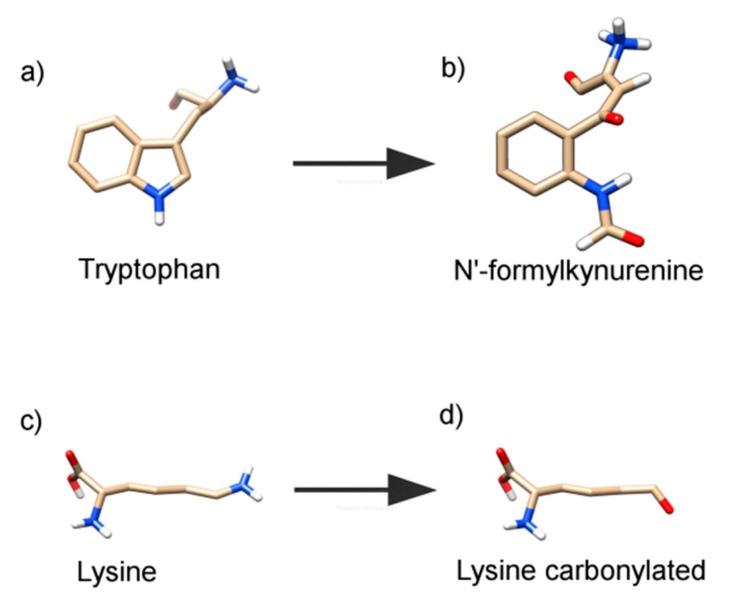
Transformation of residues. Tridimensional representation of residues changing from (**a**) tryptophan to (**b**) *N*′-formylkynurenine, and from (**c**) lysine to (**d**) carbonylated lysine. Image prepared using UCSF Chimera.

**Figure 8:**
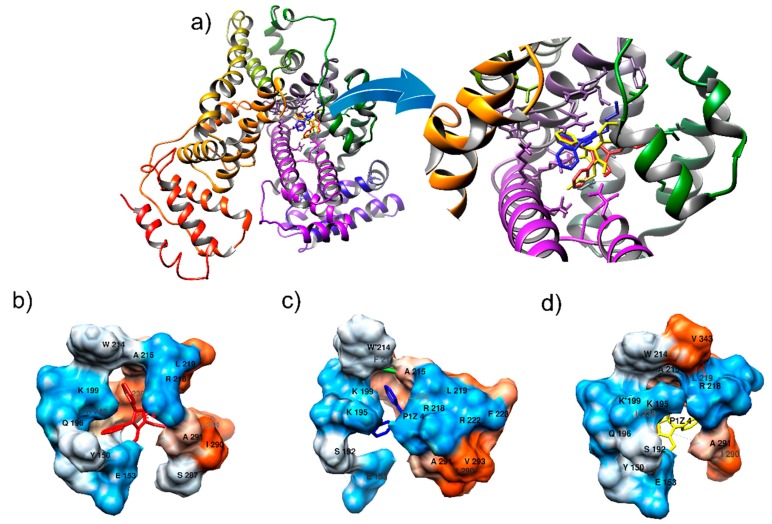
Molecular docking of native and altered HSA with phenylbutazone. (**a**) Representation of all docking conformations superimposed. The phenylbutazone conformations with lowest Amber score energy for HSA (**b**), HSA with Trp-214 altered to *N*′-formylkynurenine (**c**), and HSA with Lys-199 altered to its carbonylated form (**d**). Figure 8a including the inset shows the ligand position for these forms in red, blue, and yellow, respectively. The interaction energies calculated between phenylbutazone and HSA were −21.3 kJ/mol, −28.4 kJ/mol, and −33.9 kJ/mol, respectively. Figure 8b–d show the residues (surface model) interacting with the ligand. The HSA conformations were aligned, and the residues were labeled in black with residue letter and number, and colored using a scale from orange (hydrophobic) to blue (hydrophilic). The symbol * identifies the location of altered residues location. Image prepared using UCSF Chimera.

Based on experimental findings, we hypothesized that the tryptophan residue was converted to *N*′-formylkynurenine and the lysine residue was converted to its carbonylated form, as presented in Figure 7, which shows the tridimensional representations of these transformations. Therefore, to simulate these oxidative alterations, the protein was modeled using the Avogadro software (Version 1.2.0. It is usually cited by http://avogadro.cc/) [40], where HSA oxidized by Tau-NBr_2_ was modeled by replacing the Trp-214 with *N*′-formylkynurenine and Lys-199 was replaced with its carbonylated form. The molecular docking showed only a slight variation in phenylbutazone orientation (Figure 8), but a significant energy variation. Based on the Amber score function, while native HSA presented an interaction energy of −21.3 kJ/mol, HSA with Trp-214 altered to *N*′-formylkynurenine resulted in an energy of −28.4 kJ/mol, and HSA with Lys-199 altered to its carbonylated form resulted in an energy of −33.9 kJ/mol. This substantial decrease in energy could explain the stabilization of the ligand in the protein oxidized by Tau-NBr_2_. As expected, molecular dynamics (MD) simulations, using the re-docking selected conformation, did not show significant modifications in the interaction map. The Amber force field was employed, with implicit (re-docking) and explicit (MD simulations) solvents, validating the minimal energy results. During 10 ns of simulation with explicit solvent, it was not possible to identify any specific interactions intermediated by water. It is also important to emphasize the mostly hydrophobic characteristic of the binding region.

**Figure 9:**
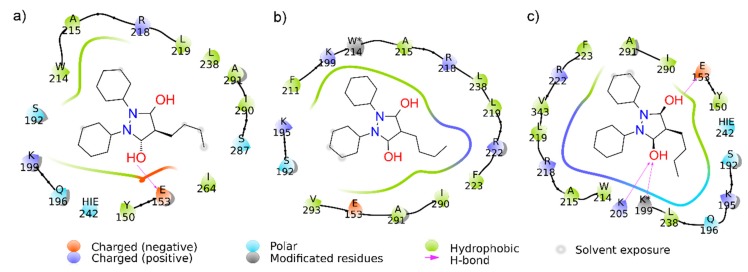
Maps of interaction. Representation of the interactions between phenylbutazone and (**a**) HSA, (**b**) HSA with Trp-214 altered to *N*′-formylkynurenine (W*214), and (**c**) HSA with Lys-199 altered to its carbonylated form (K*199). The legend at the bottom presents all types of interactions evaluated by the program Maestro, with, in particular, hydrogen bonds represented by pink arrows and hydrophobic interactions in green. The measurements of distances and orientations of the side chains for the conformation in Figure 9c suggest that only the hydrogen bond involving Lys-199 is occurring (the interaction with Lys-205 is only presented due to Maestro’s algorithm instructions). Representations prepared using Maestro (Schrödinger Release 2017-2 version 11.2.014: Maestro, Schrödinger, LLC, New York, NY, USA, 2017).

Figure 9 shows the map of the interactions for all cases evaluated, where it is possible to identify different mechanisms of interaction. The oxidized HSA model where Trp-214 was replaced by *N*′-formylkynurenine (Figure 9b) shows an increase in stabilization, mainly due to the hydrophobic interactions, differentiating it from native HSA which has one hydrogen bond (H-bond) involving residue Glu-193 (Figure 9a). The analysis of the HSA model where Lys-199 was replaced by its carbonylated form (Figure 9c) also reveals a higher stabilization of phenylbutazone when compared to native HSA. In this case, the identified interaction pattern is mainly controlled by H-bond interactions, also involving the modified residue, as well as salt bridges. A careful analysis indicates that the interaction map cannot be precise, since Lys-202 and Lys*-199 cannot perform H-bonds with the same carboxyl group at the same time. However, interactions stabilized by H-bonds suggest a water requirement, consequently facilitating the access of water to the site. This may be enough to interfere with the constant association. It is worthy of note that, for easy viewing and identification of transformed residues, their names in the figures were not modified, and the symbol * was included.

## 3. Discussion

At site I of HSA, Leu-219, Leu-238, Leu-260, Ala-261, Ala-291, Tyr-150, Arg-257, Arg-222, Lys-199, Ser-289, Ile-290, His-242, Phe-223, and Trp-214 are among the amino-acid residues most cited in complexation with ligands [17,29,30,31]. In general, hydrophobic forces, van der Waals interactions, hydrogen bonds, and salt-bridge interactions among ligands and amino-acid residues are involved in the complexation. Special attention in this work is deserving of the presence and significant contribution of Trp-214 and Lys-199, which are amino acids susceptible to oxidation in this site. 

Here, strong evidence was obtained that Tau-NBr_2_ is an excellent chemical to deplete the only tryptophan residue of HSA, without, however, provoking the denaturation of the protein. This chemical feature of Tau-NBr_2_ is related to its intermediate reactivity when compared to other strong and weak oxidizing agents of the same family, such as HOBr, HOCl, and Tau-NHCl. Indeed, a comparison of rate constants among these compounds and several biological targets provide the following general sequence: HOBr > HOCl > Tau-NBr_2_ > Tau-NHCl [32]. Specifically, a comparison between HOCl and Tau-NBr_2_ showed that, while the depletion of tryptophan residues in lysozyme was significantly higher using Tau-NBr_2_, the degradation of the protein, evidenced by its aggregation, was only observed using HOCl [41]. Here, this chemical feature of Tau-NBr_2_ was confirmed using spectroscopic and chromatographic approaches. All the results pointed to the significant consumption of tryptophan. In the context of putative alterations of HSA, evidence was provided that other non-oxidizable amino acids involved in the binding at site I did not interfere in the oxidation of Trp-214. Further evidence of the oxidation of Trp-214 in HSA was the appearance of an absorbance band centered at 315 nm, and a maximum of fluorescence at 450 nm. This spectroscopic feature is characteristic of the formation of kynurenine derivatives, the degradation product of tryptophan [36,42].

The comparison of Tau-NBr_2_ with Tau-NHCl was elucidative. As well established, Tau-NHCl is a very mild oxidant. As such, it shows high selectivity to sulfhydryl residues in proteins [43]. This property was also confirmed here, since Tau-NHCl was much more effective in the depletion of sulfhydryl residues in HSA compared to Tau-NBr_2_. This result makes sense, because as the stronger oxidant, Tau-NBr_2_ was partially consumed by the other oxidizable amino acids, i.e., Trp-214, for instance. It is worthy of note that the commercial source of HSA used here was partially oxidized, as can be concluded from the amount of sulfhydryl residues detected in the control, i.e., 3 nmol/mg (equivalent to 0.2 mol/mol, 20%). There was additional evidence that the free cysteine residue in HSA (Cys-34) was not relevant regarding the binding of phenylbutazone.

The oxidation capacity of Tau-NHCl and Tau-NBr_2_ was also evaluated by the generation of carbonyl products, a well-established marker of protein oxidation [44]. It was demonstrated that Tau-NBr_2_ was much more effective in the generation of carbonyl products in HSA compared to the oxidation by Tau-NHCl. Lys-199 is among the amino acids involved in the intramolecular forces that stabilize ligands at site I [17,29,31]. As lysine is highly susceptible to carbonylation [45], the obtained alteration in the binding capacity provoked by Tau-NBr_2_ cannot exclude the involvement of the modification of this amino-acid residue. In summary, we propose that the oxidation of Trp-214 and Lys-199 might be the main oxidative alterations provoked by Tau-NBr_2_ at site I of HSA. Obviously, this does not mean that other lysine residues in the protein could not be oxidized and also contribute to the alteration in the binding of phenylbutazone.

The alteration in the binding capacity of phenylbutazone was measured using an analytical procedure insensitive to the presence of tryptophan in the protein. This is an important issue, because the measurement of association constants is usually performed by the quenching effect of the ligands in the intrinsic fluorescence of the protein, for which tryptophan residues are the major contributor [14,46]. As this fluorescence band was depleted by Tau-NBr_2_, the application of this assay was impeded. This was the reason for choosing phenylbutazone as a representative ligand of site I in HSA. This pharmaceutical drug is susceptible to the induction of chirality when bound in the protein [47]. The CD signal generated due to the attachment of the optically inactive phenylbutazone inside the asymmetric microenvironment is a quite specific analytical signal and devoid of any absorbance or fluorescence interferences [48]. Considering that the protein and the ligand do not have circular dichroism signals in the spectral region studied, the appearance of the CD signal is direct evidence of the complexation between the protein and phenylbutazone. From this phenomenon, the association constant was measured using the increase in the CD signal from a concentration-dependent curve [39]. The association constant for the native protein (1.1 × 10^4^ M^−1^) was of the same magnitude compared to that obtained by fluorescence quenching experiments (0.29 × 10^4^ M^−1^), as reported by Maciążek-Jurczyk [49]. Corroborant with our expectation, the association constant between phenylbutazone and HSA was altered when the protein was oxidized by Tau-NBr_2_, but not by Tau-NHCl. In short, these results are evidence that the depletion of Trp-214 and the putative oxidation of Lys-199 at site I of HSA may alter the physiological function of this protein, i.e., its action as a drug-carrier. These results are in agreement with those reported by Maciazek-Jurczyk et al. [25] These authors reported ^1^H-NMR evidence of alteration of Trp-214 depletion when HSA was oxidized by chloramine-T. In agreement, the authors also reported an increase in binding constant with methotrexate, an anticancer drug.

The experimental findings were corroborated by computational simulation and revealed an increase in the interaction energy between phenylbutazone and the HSA oxidized model compared to its native form. However, the reasons for stabilization were distinct, revealing different environmental conditions in site I before and after the simulated alteration. A more hydrophobic cavity, as presented in HSA where Trp-214 was replaced by *N*′-formylkynurenine, may represent a regime of fluctuations that hinder water access, especially in the buried site. This putative obstruction of water access may contribute to the increase in association constant. It is important to note that site I is not an exposed cavity on the HSA surface when evaluated in its crystallographic structure. We cannot discard discrete variations in the conformation of secondary structure of the protein, a hypothesis that cannot be verified with the computational approaches employed here, but which could contribute to understanding the stabilization process and the increase in association constant, as obtained experimentally. Regarding the alteration provoked by substitution of Lys-199 with its carbonylated form, we highlighted the modifications in H-bond interactions compared to the native protein. In this case, the alteration in stabilization energy might be linked to enthalpic factors, which are usually related to the formation of these types of intermolecular forces [50]. The mechanism in this case may favor a dynamical regime that allows the access of water to the binding site, with higher fluctuations and a higher exposure of the cavity. Indeed, some authors [51,52], analyzing interactions between HSA (site I) and other species, reported similar conclusions about how the interactions can be enthalpically and entropically driven, and how they can affect the local regime of fluctuations of HSA, supporting the mechanisms proposed as suitable for the increase in stabilization.

## 4. Materials and Methods

### 4.1. Chemicals and Reagents

Human serum albumin (HSA), essentially fatty acid free (A1887), phenylbutazone, taurine, *N*-acetyl-l-tryptophan, *N*-acetyl-l-tyrosine, methionine, cysteine, phenylalanine, alanine, serine, arginine, leucine, 5,5′-dithiobis-(2-nitrobenzoic acid) (DTNB), and 2,4-dinitrophenylhydrazine were purchased from Sigma-Aldrich (St. Louis, MO, USA). HSA was dissolved in 10 mmol·L^−1^ phosphate-buffered saline (PBS) at pH 7.4 to give a 1 mmol·L^−1^ stock solution, and its concentration was measured by its absorbance (ε_280 nm_ = 35,219 mol^−1^·L·cm^−1^). Hypochlorous acid (HOCl) was prepared by diluting a 5% stock solution, and the concentration was determined spectrophotometrically after dilution in 0.01 mol·L^−1^ NaOH, pH 12 (ε_292 nm_ = 350 mol^−1^·L·cm^−1^). Taurine monochloramine (Tau-NHCl) was prepared by the addition of 5 mmol·L^−1^ HOCl to 50 mmol·L^−1^ taurine in PBS at pH 7.4 [53]. Hypobromous acid (HOBr) was synthesized by combining 100 mmol·L^−1^ HOCl and 200 mmol·L^−1^ NaBr in water. Taurine dibromamine (Tau-NBr_2_) was prepared by the addition of 5 mmol·L^−1^ HOBr to 2.5 mmol·L^−1^ taurine in PBS at pH 7.4 [54]. A Perkin Elmer Lambda 35 UV–visible (UV–Vis) spectrophotometer (Shelton, CT, USA) was used for the UV–Vis measurements.

### 4.2. Oxidation of N-acetyl-l-tryptophan and N-acetyl-l-tyrosine

The reaction mixtures were composed of 50 µmol·L^−1^ *N*-acetyl-l-tryptophan (*N*acTrp) or *N*-acetyl-l-tyrosine (*N*acTyr) and 500 µmol·L^−1^ Tau-NBr_2_ or 1000 µmol·L^−1^ Tau-NHCl in 10 mmol·L^−1^ PBS, pH 7.4, at 25 °C. When present, the competitor amino acids were added at 500 µmol·L^−1^. It is worthy of note that *N*acTrp and *N*acTyr were used instead tryptophan and tyrosine, due to their higher similarity with the amino-acid residues in proteins. It must also be remembered that HSA has one tryptophan, 18 tyrosines, six methionines, and 35 cysteines, with 34 in an oxidized (disulfide bond) form and one in a reduced form. Therefore, the studies were performed using at least 10-fold molar excess of the competitor amino acids to the target. The kinetics of *N*acTrp depletion were monitored using a single-mixing stopped-flow system equipped with a high-intensity light-emitting diode (LED) source (280 nm) and a cut-off filter (325 nm) (SX20/LED Stopped-Flow System, Applied Photophysics, UK). The depletion of *N*acTyr was monitored using a conventional fluorimeter (LS55 spectrofluorimeter, Perkin-Elmer, Waltham, MA, USA) with the following settings: excitation at 280 nm and emission at 315 nm, 5-nm slit widths for both excitation and emission wavelengths, and a 3-mL quartz cuvette with a 10-mm path length magnetically stirred during the measurements. The consumption of *N*acTrp was also chromatographically evaluated by high-performance liquid chromatography (HPLC; Jasco, Easton, MD, USA) in line with a fluorescence detector set at 295/350 nm. The analyses were carried out isocratically on a C18 reversed-phase column (150 mm × 4.6 mm, 4 µm, Luna, Phenomenex, Torrance, CA, USA.), with 0.1% formic acid in water and 0.1% formic acid in acetonitrile (80:20, *v*:*v*) as the mobile phase at a flow rate of 1.0 mL·min^−1^. The consumption of *N*acTyr was analyzed under the same conditions, but the detector was set at 280/310 nm, and the mobile phase was set at 90:10. For the HPLC studies, the reactions were incubated for 1 h before injections.

### 4.3. Oxidation of HSA

The reaction mixtures were composed of 10 µmol·L^−1^ HSA and 100 µmol·L^−1^ Tau-NBr_2_ or 200 µmol·L^−1^ Tau-NHCl in 10 mmol·L^−1^ PBS at 25 °C and incubated for 1 h. The intrinsic fluorescence of HSA was measured at an excitation wavelength of 295 nm, with emission in the range of 310–450 nm, 5-nm slit widths for both excitation and emission wavelengths, and a 3-mL quartz cuvette with a 10-mm path length magnetically stirred during the measurements [33]. The synchronous fluorescence spectra were obtained by scanning simultaneously with a fixed wavelength between the excitation and emission monochromators. Specifically, by fixing a delta wavelength (emission minus excitation) at 15 or 60 nm, alterations in the microenvironments of tyrosine or tryptophan, respectively, could be detected [34]. The reactions were also studied using HPLC. In these cases, the analyses were carried out on a C18 reversed-phase column (150 mm × 4.6 mm, 4 µm, Jupiter, Phenomenex). The mobile phase was solvent A (0.1% trifluoracetic acid in water) and solvent B (0.1% trifluoracetic acid in acetonitrile). The gradient was 90–40% solvent A in 20 min at a flow rate of 1.0 mL·min^−1^. The fluorescence detector was set at 295/340 nm.

### 4.4. Determination of Sulfhydryl Residues

The relative concentration of sulfhydryl residues in HSA was measured using Ellman’s method with modifications [55]. The reaction mixtures (1000 µL) were composed of 50 µmol·L^−1^ HSA and 500 µmol·L^−1^ Tau-NBr_2_ or 1000 µmol·L^−1^ Tau-NHCl in 10 mmol·L^−1^ PBS at 25 °C and incubated for 1 h; then, 1000 µmol·L^−1^ methionine was added to scavenge the remaining oxidants. The reaction mixtures were incubated for an additional 10 min before measurement of sulfhydryl residues. Then, 250 µL of 100 mmol·L^−1^ borate buffer (pH 8.2) was added, followed by 25 µL of a 10 mmol·L^−1^ working solution of DTNB. The absorbance was measured at 412 nm. The results were presented in nmol of sulfhydryl residues per mg of protein.

### 4.5. Determination of Carbonyl Products

The content of carbonyl products was measured as described previously with modifications [56]. The reaction mixtures (200 µL) were composed of 50 µmol·L^−1^ HSA and 500 µmol·L^−1^ Tau-NBr_2_ or 1000 µmol·L^−1^ Tau-NHCl in 10 mmol·L^−1^ PBS at 25 °C and incubated for 1 h; then, 1000 µmol·L^−1^ methionine was added to scavenge the remaining oxidants. The reaction mixtures were incubated for an additional 10 min before measurement of carbonyl products. Then, 2 mL of 2.5 mmol·L^−1^ 2,4-dinitrophenylhydrazine dissolved in 2.5 mol·L^−1^ HCl was added to each sample, with the control having only 2.5 mol·L^−1^ HCl. After 1 h, cold 20% trichloroacetic acid was added, and the samples were kept in the refrigerator for 10 min, and then centrifuged at 3000 rpm for 10 min. The protein pellet was washed three times with a mixture of ethyl acetate–ethanol 1:1 for the extraction of lipids and unreacted 2,4-dinitrophenylhydrazine. The pellet was dried to remove the solvent and dissolved in 2 mL of 8.0 mol·L^−1^ urea. The mixture was maintained in a boiling water bath until complete dissolution of the pellet. The absorbance of the solutions was measured at 370 nm. The amount of carbonyl groups was estimated from the difference between optical densities of the experimental and control samples at 370 nm, and was represented in nmol of carbonyl product per mg of protein. The molar extinction coefficient was 22,000 mol^−1^·L·cm^−1^.

### 4.6. Determination of Association Constant

The binding of phenylbutazone with HSA was studied by the induction of chirality (∆mdeg at 292 nm) in this drug provoked by its attachment within the cavities of the protein [48]. The reaction mixtures were composed of 30 µmol·L^−1^ HSA and 300 µmol·L^−1^ Tau-NBr_2_ or 600 µmol·L^−1^ Tau-NHCl in 10 mmol·L^−1^ PBS at 25 °C and incubated for 1 h; then, 1000 µmol·L^−1^ methionine was added to scavenge the remaining oxidants, and the reaction mixtures incubated for additional 10 min before titration with phenylbutazone (0–90 µmol·L^−1^). Before measurements, the mixture was incubated for 2 min. The circular dichroism studies were performed in a Jasco J-815 spectropolarimeter (Jasco, Japan) equipped with a thermostatically controlled cell holder. The spectra were obtained with 1-nm-step resolution, a response time of 1 s, and a scanning speed of 50 nm/min. A 3-mL quartz cuvette with a 10-mm path length and a magnetic stirrer were used for the measurements. The baseline (PBS) was subtracted from all measurements. The association constant was calculated by non-linear fittings using GraphPad Prism version 5.00 for Windows (GraphPad Software, San Diego, CA, USA).

### 4.7. Molecular Docking Simulations

The interactions between the receptor (HSA) and the ligand (phenylbutazone) were evaluated through molecular docking computational tools. The receptor and ligand were obtained from the protein data bank (PDB) at www.rcsb.org (accessed on: 9 July 2017) [57] deposited with the PDB identifier 2BXC [17]. In preparation for the docking process, chain A of HSA complexed with phenylbutazone was selected from the PDB structure, solvated, equilibrated, and relaxed through molecular dynamics using the tool “Molecular Dynamics Simulations” of “MD/Ensemble Analysis” from UCSF Chimera version 1.12 [58]. The simulation employed a box with a distance of 10 Å from the outermost residue of the protein surface and the box edge. The system was solvated with the transferable intermolecular potential with three points (TIP3P; TIP3PBOX) water model and ions of Na^+^ were included to neutralize the net charge (23,912 solvent molecules and ions included). The minimization process was composed by 50,000 steepest-descent steps followed by 1000 conjugate-gradient steps, both using a 0.01-Å-size step. The equilibration employed 50,000 steps using the velocity scaled based on a temperature of 298 K, with the thermostat applied every 2 fs and with a time step of 2 fs. The production ran 5,000,000 steps starting from the equilibration with a Nosé–Hoover thermostat at 298 K and a relaxation time of 0.2 fs, with a time step of 2 fs. The dominant conformation was obtained from clustering with the “Dynamics Trajectory” analysis tool. The oxidation of HSA was modeled using the Avogadro software, where HSA oxidized by Tau-NBr_2_ was modeled by replacing Trp-214 with *N*′-formylkynurenine, and by replacing Lys-199 with its carbonylated form. Only chain A of HSA was employed as a receptor, and was prepared for the simulations using UCSF Chimera. The preparation involved the addition of hydrogen and the calculation of charges, where Amber force field ff99sb was employed for standard residues and AM1-BCC was employed for non-standard residues. The same procedures were applied to the ligand, where Antechamber [59] was used for the generation of the topology file. UCSF Dock 6 was used for the generation of the topology file. The molecular docking simulations were carried out using UCSF Dock 6 [60], where the binding cavities were selected based on HSA site I. UCSF Dock 6 was chosen based on the docking success evaluations for reconstructing the dominant complex conformation. The complete molecular docking calculations were composed by docking scores using the Grid Score function and a re-docking with Amber Score. The initial docking used a rigid receptor and flexible ligand setting of up to 0.2 Å for the orientations of the ligand, receptor, and overlap bins, and with a distance tolerance for matching of 0.75 Å. Dock 6 used the Anchor and Grow algorithm configured to evaluate 500 conformations, 1500 times per receptor (750,000 evaluations). During the re-docking process, the protein and ligand are flexible; thus, the residues near the ligand and the ligand itself are movable, while the distant atoms are kept frozen. The rescoring with Amber Score function employs the implemented Generalized Born surface area continuum model for solvation free energy, which is as accurate a method as explicit solvent models within the Amber Force field for ranking the energy. The conformation with the lowest Amber score energy for each receptor was selected. The molecular docking representations and analyses were performed using UCSF Chimera and Schrödinger Maestro (Schrödinger Release 2017-2 version 11.2.014: Maestro, Schrödinger, LLC, NY, USA, 2017).

## 5. Conclusions

We demonstrated that the mild oxidant Tau-NBr_2_ was able to deplete the only tryptophan residue and was able to promote the carbonylation of HSA. These findings are relevant since the oxidative modification of amino-acid residues in HSA may influence the ability of the protein as a drug carrier [21]. As well established, HOCl and HOBr are produced by leukocytes, and taurine is the most abundant free amino acid in these cells; the produced haloamines can diffuse long distances and cross the plasma membrane [61]. In other words, it can be conceived that the oxidative alterations studied here are, indeed, plausible to occur in vivo. Using phenylbutazone, we demonstrated this effect. The experimental findings were corroborated by computational simulation, where Trp-214 was altered to *N*′-formylkynurenine, and Lys-199 was altered to its carbonylated form. These results strengthen the proposal that oxidative stress may alter the physiological properties of HSA. Particularly, our results show that its capacity as a site-I drug carrier can be directly affected, consequently altering the pharmacokinetic properties of these drugs.

## References

[1] Pisoschi A.M., Pop A. (2015). The role of antioxidants in the chemistry of oxidative stress: A review. Eur. J. Med. Chem..

[2] Araújo R.F.F., Martins D.B.G., Borba M.A.C.S.M., Morales-Gonzales J.A., Morales-Gonzales A., Madrigal-Santillan E.O. (2016). Oxidative Stress and Disease. A Master Regulator of Oxidative Stress-The Transcription Factor Nrf2.

[3] Poprac P., Jomova K., Simunkova M., Kollar V., Rhodes C.J., Valko M. (2017). Targeting Free Radicals in Oxidative Stress-Related Human Diseases. Trends Pharmacol. Sci..

[4] Moloney J.N., Cotter T.G. (2018). ROS signalling in the biology of cancer. Semin. Cell Dev. Biol..

[5] Rani V., Deep G., Singh R.K., Palle K., Yadav U.C.S. (2016). Oxidative stress and metabolic disorders: Pathogenesis and therapeutic strategies. Life Sci..

[6] Neves Carvalho A., Firuzi O., Joao Gama M., van Horssen J., Saso L. (2017). Oxidative Stress and Antioxidants in Neurological Diseases: Is There Still Hope?. Curr. Drug Targets.

[7] Tramutola A., Lanzillotta C., Perluigi M., Butterfield D.A. (2017). Oxidative stress, protein modification and Alzheimer disease. Brain Res. Bull..

[8] Li S., Tan H.-Y., Wang N., Zhang Z.-J., Lao L., Wong C.-W., Feng Y. (2015). The Role of Oxidative Stress and Antioxidants in Liver Diseases. Int. J. Mol. Sci..

[9] Niedzielska E., Smaga I., Gawlik M., Moniczewski A., Stankowicz P., Pera J., Filip M. (2016). Oxidative Stress in Neurodegenerative Diseases. Mol. Neurobiol..

[10] Roche M., Rondeau P., Singh N.R., Tarnus E., Bourdon E. (2008). The antioxidant properties of serum albumin. FEBS Lett..

[11] Prakash S. (2017). Role of human serum albumin and oxidative stress in diabetes. J. Appl. Biotechnol. Bioeng..

[12] Peters T. (1996). All about Albumin: Biochemistry, Genetics, and Medical Applications.

[13] Kouno Y., Anraku M., Yamasaki K., Okayama Y., Iohara D., Nakamura H., Maruyama T., Hirayama F., Kragh-Hansen U., Otagiri M. (2016). *N*-acetyl-l-methionine is a superior protectant of human serum albumin against post-translational oxidation as compared to *N*-acetyl-l-tryptophan. Biochem. Biophys. Rep..

[14] Lakowicz J.R. (2006). Principles of Fluorescence Spectroscopy.

[15] Zhivkova Z. (2015). Studies on Drug—Human Serum Albumin Binding: The Current State of the Matter. Curr. Pharm. Des..

[16] Sudlow G., Birkett D.J., Wade D.N. (1975). The characterization of two specific drug binding sites on human serum albumin. Mol. Pharmacol..

[17] Ghuman J., Zunszain P.A., Petitpas I., Bhattacharya A.A., Otagiri M., Curry S. (2005). Structural Basis of the Drug-binding Specificity of Human Serum Albumin. J. Mol. Biol..

[18] Maciążek-Jurczyk M., Szkudlarek A., Chudzik M., Pożycka J., Sułkowska A. (2018). Alteration of human serum albumin binding properties induced by modifications: A review. Spectrochim. Acta Part A Mol. Biomol. Spectrosc..

[19] Naldi M., Baldassarre M., Domenicali M., Caraceni P. (2017). Structural and functional integrity of human serum albumin: Analytical approaches and clinical relevance in patients with liver cirrhosis. J. Pharm. Biomed. Anal..

[20] Oettl K., Marsche G. (2010). Redox State of Human Serum Albumin in Terms of Cysteine-34 in Health and Disease. Methods Enzymol..

[21] Sitar M.E., Aydin S., Cakatay U. (2013). Human serum albumin and its relation with oxidative stress. Clin. Lab..

[22] Arif Z., Neelofar K., Arfat M.Y., Zaman A., Tarannum A., Parveen I., Ahmad S., Khan M.A., Badar A., Islam S.N. (2018). Hyperglycemia induced reactive species trigger structural changes in human serum albumin of type 1 diabetic subjects. Int. J. Biol. Macromol..

[23] Fujii R., Ueyama J., Aoi A., Ichino N., Osakabe K., Sugimoto K., Suzuki K., Hamajima N., Wakai K., Kondo T. (2018). Oxidized human serum albumin as a possible correlation factor for atherosclerosis in a rural Japanese population: The results of the Yakumo Study. Environ. Health Prev. Med..

[24] Maciążek-Jurczyk M., Sułkowska A. (2015). Spectroscopic analysis of the impact of oxidative stress on the structure of human serum albumin (HSA) in terms of its binding properties. Spectrochim. Acta Part A Mol. Biomol. Spectrosc..

[25] Maciążek-Jurczyk M., Sułkowska A., Równicka-Zubik J. (2016). Alteration of methotrexate binding to human serum albumin induced by oxidative stress. Spectroscopic comparative study. Spectrochim. Acta Part A Mol. Biomol. Spectrosc..

[26] Oettl K., Birner-Gruenberger R., Spindelboeck W., Stueger H.P., Dorn L., Stadlbauer V., Putz-Bankuti C., Krisper P., Graziadei I., Vogel W. (2013). Oxidative albumin damage in chronic liver failure: Relation to albumin binding capacity, liver dysfunction and survival. J. Hepatol..

[27] Russeva V.N., Zhivkova Z.D. (1999). Protein binding of some nonsteroidal anti-inflammatory drugs studied by high-performance liquid affinity chromatography. Int. J. Pharm..

[28] Ximenes V.F., da Fonseca L.M., de Almeida A.C. (2011). Taurine bromamine: A potent oxidant of tryptophan residues in albumin. Arch. Biochem. Biophys..

[29] Zhang Y., Lee P., Liang S., Zhou Z., Wu X., Yang F., Liang H. (2015). Structural Basis of Non-Steroidal Anti-Inflammatory Drug Diclofenac Binding to Human Serum Albumin. Chem. Biol. Drug Des..

[30] Ryan A.J., Chung C.-W., Curry S. (2011). Crystallographic analysis reveals the structural basis of the high-affinity binding of iophenoxic acid to human serum albumin. BMC Struct. Biol..

[31] Ryan A.J., Ghuman J., Zunszain P.A., Chung C., Curry S. (2011). Structural basis of binding of fluorescent, site-specific dansylated amino acids to human serum albumin. J. Struct. Biol..

[32] De Carvalho Bertozo L., Morgon N., De Souza A., Ximenes V. (2016). Taurine Bromamine: Reactivity of an Endogenous and Exogenous Anti-Inflammatory and Antimicrobial Amino Acid Derivative. Biomolecules.

[33] Möller M., Denicola A. (2002). Protein tryptophan accessibility studied by fluorescence quenching. Biochem. Mol. Biol. Educ..

[34] Chen Y.-C., Wang H.-M., Niu Q.-X., Ye D.-Y., Liang G.-W. (2016). Binding between Saikosaponin C and Human Serum Albumin by Fluorescence Spectroscopy and Molecular Docking. Molecules.

[35] Reid L.O., Roman E.A., Thomas A.H., Dántola M.L. (2016). Photooxidation of Tryptophan and Tyrosine Residues in Human Serum Albumin Sensitized by Pterin: A Model for Globular Protein Photodamage in Skin. Biochemistry.

[36] Ehrenshaft M., Silva S.O., Perdivara I., Bilski P., Sik R.H., Chignell C.F., Tomer K.B., Mason R.P. (2009). Immunological detection of N-formylkynurenine in oxidized proteins. Free Radic. Biol. Med..

[37] Hong P., Koza S., Bouvier E.S.P. (2012). Size-Exclusion Chromatography for the Analysis of Protein Biotherapeutics and their Aggregates. J. Liq. Chromatogr. Relat. Technol..

[38] Fasman G.D. (1996). Circular Dichroism and the Conformational Analysis of Biomolecules.

[39] Zsila F., Bikádi Z., Simonyi M. (2003). Probing the binding of the flavonoid, quercetin to human serum albumin by circular dichroism, electronic absorption spectroscopy and molecular modelling methods. Biochem. Pharmacol..

[40] Hanwell M.D., Curtis D.E., Lonie D.C., Vandermeersch T., Zurek E., Hutchison G.R. (2012). Avogadro: An advanced semantic chemical editor, visualization, and analysis platform. J. Cheminform..

[41] Petrônio M.S., Ximenes V.F. (2012). Effects of oxidation of lysozyme by hypohalous acids and haloamines on enzymatic activity and aggregation. Biochim. Biophys. Acta.

[42] Fukunaga Y., Katsuragi Y., Izumi T., Sakiyama F. (1982). Fluorescence characteristics of kynurenine and *N*′-formylkynurenine. Their use as reporters of the environment of tryptophan 62 in hen egg-white lysozyme. J. Biochem..

[43] Grigoryan H., Li H., Iavarone A.T., Williams E.R., Rappaport S.M. (2012). Cys34 Adducts of Reactive Oxygen Species in Human Serum Albumin. Chem. Res. Toxicol..

[44] Dalle-Donne I., Rossi R., Giustarini D., Milzani A., Colombo R. (2003). Protein carbonyl groups as biomarkers of oxidative stress. Clin. Chim. Acta.

[45] Khosravifarsani M., Monfared A.S., Pouramir M., Zabihi E. (2016). Effects of Fenton Reaction on Human Serum Albumin: An In Vitro Study. Electron. Physician.

[46] Ghisaidoobe A., Chung S. (2014). Intrinsic Tryptophan Fluorescence in the Detection and Analysis of Proteins: A Focus on Förster Resonance Energy Transfer Techniques. Int. J. Mol. Sci..

[47] Tedesco D., Bertucci C. (2015). Induced circular dichroism as a tool to investigate the binding of drugs to carrier proteins: Classic approaches and new trends. J. Pharm. Biomed. Anal..

[48] Ascoli G., Bertucci C., Salvadori P. (1995). Stereospecific and competitive binding of drugs to human serum albumin: A difference circular dichroism approach. J. Pharm. Sci..

[49] Maciążek-Jurczyk M. (2014). Phenylbutazone and ketoprofen binding to serum albumin. Fluorescence study. Pharmacol. Rep..

[50] Lazaridis T., Archontis G., Karplus M. (1995). Enthalpic Contribution to Protein Stability: Insights from Atom-Based Calculations and Statistical Mechanics. Adv. Protein Chem..

[51] Jones C.L., Dickson T., Hayes R., Thomas L. (2012). Effects of pH and ionic strength on the thermodynamics of human serum albumin-photosensitizer binding. Thermochim. Acta.

[52] Li Y., Yan X.-P., Chen C., Xia Y.-L., Jiang Y. (2007). Human Serum Albumin−Mercurial Species Interactions. J. Proteome Res..

[53] Tokunaga S., Kanayama A., Miyamoto Y. (2007). Modification of IκBα by taurine bromamine inhibits tumor necrosis factor α-induced NF-κB activation. Inflamm. Res..

[54] Thomas E.L., Bozeman P.M., Jefferson M.M., King C.C. (1995). Oxidation of bromide by the human leukocyte enzymes myeloperoxidase and eosinophil peroxidase. Formation of bromamines. J. Biol. Chem..

[55] Riener C.K., Kada G., Gruber H.J. (2002). Quick measurement of protein sulfhydryls with Ellman’s reagent and with 4,4′-dithiodipyridine. Anal. Bioanal. Chem..

[56] Azizova O.A., Aseychev A.V., Beckman E.M., Moskvina S.N., Skotnikova O.I., Smolina N.V., Gryzunov Y.A., Dobretsov G.E. (2012). Studies of oxidant-induced changes in albumin transport function with a fluorescent probe k-35. Effect of hypochlorite. Bull. Exp. Biol. Med..

[57] Berman H.M., Westbrook J., Feng Z., Gilliland G., Bhat T.N., Weissig H., Shindyalov I.N., Bourne P.E. (2000). The Protein Data Bank. Nucleic Acids Res..

[58] Pettersen E.F., Goddard T.D., Huang C.C., Couch G.S., Greenblatt D.M., Meng E.C., Ferrin T.E. (2004). UCSF Chimera?A visualization system for exploratory research and analysis. J. Comput. Chem..

[59] Wang J., Wang W., Kollman P.A., Case D.A. (2006). Automatic atom type and bond type perception in molecular mechanical calculations. J. Mol. Graph. Model..

[60] Lang P.T., Brozell S.R., Mukherjee S., Pettersen E.F., Meng E.C., Thomas V., Rizzo R.C., Case D.A., James T.L., Kuntz I.D. (2009). DOCK 6: Combining techniques to model RNA-small molecule complexes. RNA.

[61] Asahi T., Nakamura Y., Kato Y., Osawa T. (2015). Specific role of taurine in the 8-brominated-2′-deoxyguanosine formation. Arch. Biochem. Biophys..

